# Can machine learning algorithms perform better than multiple linear regression in predicting nitrogen excretion from lactating dairy cows

**DOI:** 10.1038/s41598-022-16490-y

**Published:** 2022-07-21

**Authors:** Xianjiang Chen, Huiru Zheng, Haiying Wang, Tianhai Yan

**Affiliations:** 1 Livestock Production Science Branch, Agri-Food and Biosciences Institute, Hillsborough, County Down BT26 6DR UK; 2 School of Computing, University of Ulster, Belfast, BT15 1ED UK

**Keywords:** Environmental impact, Natural hazards

## Abstract

This study aims to compare the performance of multiple linear regression and machine learning algorithms for predicting manure nitrogen excretion in lactating dairy cows, and to develop new machine learning prediction models for MN excretion. Dataset used were collated from 43 total diet digestibility studies with 951 lactating dairy cows. Prediction models for MN were developed and evaluated using MLR technique and three machine learning algorithms, artificial neural networks, random forest regression and support vector regression. The ANN model produced a lower RMSE and a higher CCC, compared to the MLR, RFR and SVR model, in the tenfold cross validation. Meanwhile, a hybrid knowledge-based and data-driven approach was developed and implemented to selecting features in this study. Results showed that the performance of ANN models were greatly improved by the turning process of selection of features and learning algorithms. The proposed new ANN models for prediction of MN were developed using nitrogen intake as the primary predictor. Alternative models were also developed based on live weight and milk yield for use in the condition where nitrogen intake data are not available (e.g., in some commercial farms). These new models provide benchmark information for prediction and mitigation of nitrogen excretion under typical dairy production conditions managed within grassland-based dairy systems.

## Introduction

Dairy cows do not efficiently utilize dietary nitrogen (N), primarily in the form of protein, and excrete a large proportion of dietary N to environment, causing terrestrial eutrophication, biodiversity loses and soil acidification^1–3^. In addition to the environmental pollution, N-related pollutants (e.g. ammonia) are linked to lung diseases, chronic bronchitis and premature mortality^4^. In Europe, approximately 75% of ammonia emitted to the atmosphere comes from livestock production^5^. Furthermore, protein supplements are the most expensive ingredient in dairy cows’ rations, so N excretion represents an economic loss. As a consequence, economic and environmental pressures are focusing attention on reducing manure N (MN) excretion from dairy production systems. Therefore, it is critical for dairy production industry to have capacity to accurately predict/mitigate MN excretion, in order to enhance economic stability and reduce environmental impacts of dairy farming.

Multiple linear regression (MLR) analysis is one of widely used modelling approaches for evaluation of MN excretion from livestock production. So far, a large number of statistical models, principally based on linear regression and MLR, have been established to predict MN excretion from dairy cows^6,7^. These studies found that equations had higher prediction accuracy for MN when using dietary variables, e.g., N intake (NI), dietary forage proportion (FP) and dietary N content (DNC), and animal factors, e.g., live weight (LW), milk yield (MY) and days in milk, as predictors^1,6,7^. However, MLR analysis makes four principal assumptions: the linearity of the relationship between dependent and independent variables, statistical independence of the errors, homoscedasticity of the errors and normality of the error distribution^8,9^. A challenge in implementing MLR technique is that these assumptions may not always be fulfilled, which might lead to biased results and fail to provide satisfactory prediction.

However, machine learning algorithms are quite beneficial when handling non-linear and complex datasets without any prior assumption, even if datasets are noisy and imprecise^10^, make machine learning algorithms, e.g. artificial neural networks (ANN), random forest regression (RFR), support vector regression (SVR), appropriate candidates to explore deep relationships between resource inputs and product outputs in livestock production. For example, Chen et al.^11^ found that ANN model had a better performance than MLR model in prediction of dairy cattle manure nutrient concentration, although Craninx et al.^10^ did not observe a better performance by ANN models when compared to MLR models in evaluation of relationships between rumen fermentation pattern and milk fatty acids in dairy cows. The RFR model made more accurate prediction than MLR model in prediction of individual survival rates to second lactation in Holstein cattle^12^. Faridi et al.^13^ evaluated the performance of SVR models and neural network models for predicting bodyweight and carcass weight of broiler chicken and found that the SVR method achieved better accuracy and generalization than the neural network method. These results imply that machine learning algorithms might be a better alternative, rather than MLR, to develop robust models for prediction of MN in cattle production. This is because N excretion rates in cattle are regulated by many animal and dietary factors (e.g., LW, productivity, feed intake and dietary N and fiber concentrations) and the interaction between these factors^14,15^. Machine learning algorithms may have technical power to explore and identify the deep and complexed relationships of N excretion rates against animal/dietary factors and their interaction effects. However, there is little information available on using machine learning algorithms to explore relationships between dairy cow MN and animal and dietary factors. Therefore, this study was designed to address this knowledge gap by using total diet digestibility data of lactating dairy cows to compare the predictive performance of different machine learning algorithms with MLR approach in predicting manure N excretion, and then develop new machine learning models for accurate prediction of MN for dairy production.

## Results and discussion

### Comparison of prediction performance of MN between MLR and machine learning models

There is little information available in the literature on the evaluation of prediction performance for MN excretion of dairy cows using MLR models against machine learning algorithms. Therefore, the research framework (the first objective) of the present study started with comparing predictive ability of machine learning approaches (ANN, SVR and RFR) against a typical MLR model published in 2006 (Yan et al.^6^) for prediction of MN output of dairy cows.

### Feature selection

**Figure 1:**
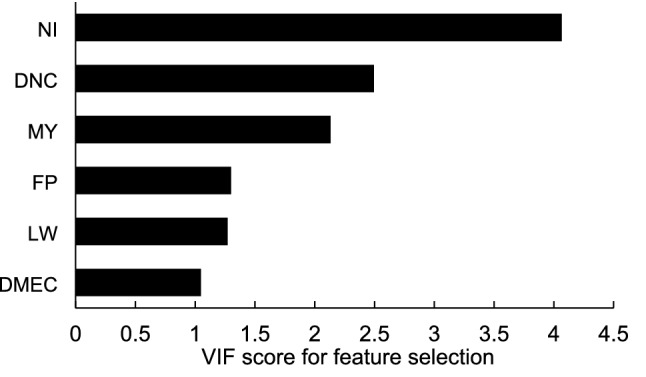
The variance inflation factors (VIF) score of features selected based on the training dataset. The features included N intake (NI), diet N content (DNC), milk yield (MY), forage proportion (FP), live weight (LW) and diet metabolizable energy content (DMEC).

To select relevant features for machine learning models (ANN, RFR and SVR), a hybrid knowledge-based and data driven approach was developed and implemented in this study. Based on Pearson correlation matrix and VIF technique, 6 features with the VIF scores lower than 5 and these features were selected as input features to model manure N output from lactating dairy cows using the present training dataset (Fig. 1). The features selected were NI, DNC, MY, FP, LW and DMEC (diet metabolizable energy content). The DMEC had the lowest VIF score (1.1) among those features. Three of those features (NI, LW, MY) were included in the MLR model of Yan et al.^6^ which was used as benchmark model in the present study. A range of prediction equations for MN output in dairy cow have been developed based on linear and multiple linear regression with stepwise procedure^6,7,14,16,17^. Among these equations, NI, LW and MY were the most commonly selected predictors (features) for the prediction. The NI has been found to be a better predictor for MN output than LW or MY in dairy cows and beef cattle^7,18^. Although the relationship between MN and LW or MY was not strong, the model performance was improved significantly when using NI, LW and MY together as predictors^6^. Furthermore, in the present study, DNC, FP and DMEC were also selected as features using Pearson correlation matrix and VIF technique. This selection is consistent with the domain knowledge, i.e., the higher N concentration in dairy cow diets (DNC), the higher N consumption (NI) and then more N excretion in manure (MN). For grassland-based dairy systems, increasing the proportion of grazed grass or silage in dairy cow diets (FP) would normally reduce NI and consequently total N excretion in manure (MN). These features (DNC, FP and DMEC) have been selected in a number of published MLR models as predictors for MN output in dairy cows^1,7,19^.

### Comparison of prediction performance of the four selected models

**Figure 2:**
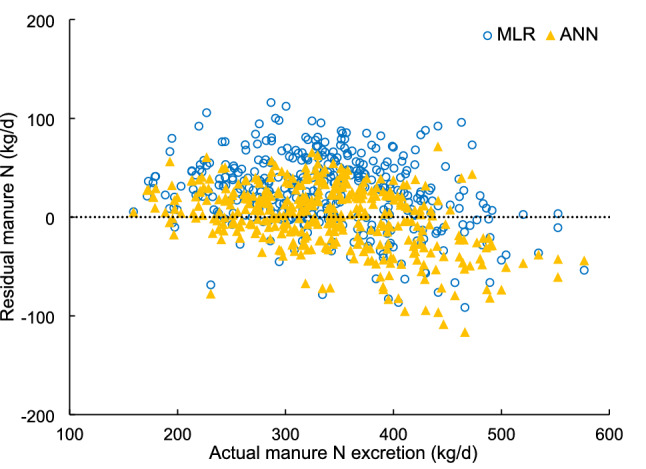
The relationship between actual and residual (predicted–actual) manure N output of dairy cows with predicted manure N performed by models developed using the multiple linear regression (MLR) and artificial neural network (ANN), respectively.

Prediction performance metrics using the present testing dataset performed by MLR, ANN, RFR and SVR models are showed in Table 1. The root mean square error (RMSE) was selected as the criteria for evaluation of the precision of models. The concordance correlation coefficient (CCC) was used to assess agreement between observed and predicted values. The CCC represents both the accuracy and precision of model performance, because it is calculated from the Pearson correlation coefficient multiplied by a bias-correction factor. For the prediction of MN using features selected by the Stepwise method, ANN model had a significantly the lowest RMSE (*P* < 0.01) and highest CCC when compared to MLR, RFR and SVR models. No significant differences in both RMSE and CCC were observed among MLR, RFR and SVR models. When using features selected by the VIF method, a similar result was obtained with the lowest RMSE and greatest CCC with the ANN model, which was significantly lower than those with RFR and SVR models. As RFR and SVR models had no improvement on the prediction accuracy of MLR model, a further evaluation was conducted by comparison of MLR versus ANN in relationships of residual plots (predicted–actual MN) against actual MN. The result is presented in Fig. 2. The ANN has a lower mean residual MN (0 vs. 25 kg/d) and a lower SD value (32.8 vs. 36.6) than the MLR. The majority of the plot data with the ANN model was evenly distributed around the y = 0 line (Fig. 2), but most of plot data with the MLR model was above that line. These results indicate that the ANN model could accurately predict MN excretion from dairy cows, while MLR, on average, overestimated MN excretion. This means that the ANN model was constructed successfully with a higher accuracy in the present study, when compared with the MLR model.

**Table 1:** Predictive performance of different modeling approaches for manure N output using stepwise and variance inflating factors (VIF) as feature selection methods.

Models^1^	RMSE^2^		CCC^3^	
	Stepwise^4^	VIF^5^	Stepwise^4^	VIF^5^
MLR	44.7^b^	/	0.60^ab^	/
RFR	46.8^b^	38.3^b^	0.58^a^	0.68^b^
SVR	44.9^b^	45.3^c^	0.64^b^	0.63^a^
ANN	34.7^a^	28.5^a^	0.70^c^	0.78^c^
*Sig*.^6^	*P* < 0.01	*P* < 0.01	*P* < 0.01	*P* < 0.01

^a,b,c^Means within a column with different superscripts differ (*P* < 0.05).

^1^*MLR* multiple linear regression; *RFR* random forests regression; *SVR* support vector regression; *ANN* artificial neural network.

^2^*RMSE* root mean square error (obtained by tenfold cross validation).

^3^*CCC* concordance correlation coefficients (obtained by tenfold cross validation).

^4^The features selected by using stepwise methods were NI (N intake), LW (live weight) and MY (milk yield).

^5^The features selected by using variance inflating factors (VIF) method were NI (N intake), LW (live weight), MY (milk yield), FP (forage proportion), DNC (diet N concentration) and DMEC (diet metabolizable energy concentration).

^6^The significance was determined by one-way analysis of variance and followed by Tukey’s Honest Significant Difference (HSD) test (n = 10, α = 0.05).

The ANN, RFR and SVR can be applied to approximate any complex functional relationship. These models have been applied in many studies in animal science to evaluate rumen fermentation pattern^10^, animal diet formulation^20^, and milk yield^21^. However, these models did not always perform better than MLR models. Chen et al.^11^ reported that ANN models had better performance in prediction of dairy cattle manure nutrient concentration when compared with MLR models, while Craninx et al.^10^ did not find that ANN models could perform better than MLR models in evaluation of relationships between rumen fermentation pattern and milk fatty acid profile. The RFR models had a higher prediction accuracy than MLR models in prediction of individual survival rates to the second lactation in Holstein dairy cows^12^. Hence, these results may indicate that the prediction performance of machine learning algorithms and MLR not only relates to their modelling power/capacity, but also depends on the nature of target data and relationships to be explored. Further investigation may need to explore the modelling potential of machine learning algorithms (e.g., ANN) for development of robust prediction models for mitigation of environmental footprint (e.g. MN excretion) in livestock production.

### Development of ANN models for prediction of MN output of dairy cows

The comparison of prediction performance for MN output in dairy cows in the present study indicated that the ANN model performed significantly better than the MLR model and other machine learning models. Therefore, the second objective of the present study was to establish new ANN models for more accurate prediction of MN output from lactating dairy cows.

### Artificial Neural Network model selection and turning

The ANN can provide universal and highly flexible function approximates for all kinds of data^22^. The ANN models have several factors, such as function adoption, network architecture and learning algorithms, and the application of these factors may affect the performance of ANN models. Once the features of ANN model are decided, architecture of network is determined mainly by artificial neurons numbers in the hidden layer. Therefore, selecting a suitable number of neurons in the hidden layer is important for ANN model development.

In the present study, in order to obtain the optimal architecture and parameters for development of ANN models, ANNs were trained by varying features, numbers of hidden layer(s) and neuron(s), training algorithms, learning rates and the threshold for partial derivatives of the error function as stopping criteria. A total of 39 ANN architectures were trained using the same features and various numbers of hidden layer(s) and numbers of neuron(s) in each hidden layer, with the objective to find the optimal number of hidden layer(s) and the corresponding neurons for each hidden layer (Table 2). As shown in Table 2, a change of numbers of hidden layers and neurons in each hidden layer greatly affected the performance of ANN models. One of the architectures with 2 hidden layers (3 and 6 neurons for the first and second layers, respectively) had the lowest RRMSE value. Therefore, the optimal architecture of the ANN model for prediction of MN in lactating dairy cows was a feed-forward network with 2 hidden layers, the first layer with 3, and the second one with 6 hidden neurons. Because there is no rule for the selection of numbers of hidden layer(s) and numbers of neuron(s) in the hidden layer, no similar results in terms of numbers of neuron(s) and hidden layer(s) were found in the published ANN models. For example, Craninx et al.^10^ developed an ANN model with one hidden layer and 6 hidden neurons for predicting rumen acetate, propionate and butyrate proportion. Felipe et al.^23^ used one hidden layer and 3 neurons in an ANN model for prediction of poultry egg production. In addition, Chen et al.^11^ found that the best ANN models for predicting of manure nutrient concentration were those which had one hidden layer with 7 hidden neurons for ammonium N, 12 for total potassium, 9 for total N and 8 for total phosphorus. Results obtained indicate that the process for selecting and obtaining the optimal configuration (consists of nodes in input layer, number of hidden layers and nodes in hidden layer/layers and nodes in output layer) is essential for the development of ANN models, although currently there is no standard approach for this process.

**Table 2:** Prediction accuracy of ANN model for predicting manure N output affected by network structure.

Network structure^1^	RRMSE^2^			
	Minimum	Maximum	Median	Mean
6–1(1)–1	8.40	9.01	8.80	8.54
6–1(2)–1	8.23	8.93	8.73	8.48
6–1(3)–1	8.11	8.83	8.52	8.44
6–1(4)–1	8.12	8.63	8.31	8.35
6–1(5)–1	8.14	8.95	8.32	8.40
6–1(6)–1	8.07	8.79	8.28	8.32
6–2(2,1)–1	8.29	8.60	8.49	8.46
6–2(2,2)–1	8.15	8.48	8.41	8.39
6–2(2,3)–1	8.14	8.36	8.32	8.26
6–2(2,4)–1	8.21	8.29	8.24	8.25
6–2(2,5)–1	8.24	8.84	8.32	8.36
6–2(2,6)–1	8.22	8.33	8.21	8.23
6–2(3,1)–1	8.08	8.60	8.35	8.33
6–2(3,2)–1	8.10	8.61	8.23	8.32
6–2(3,3)–1	8.17	8.64	8.41	8.39
6–2(3,4)–1	8.15	8.38	8.18	8.20
6–2(3,5)–1	8.12	8.36	8.15	8.18
6–2(3,6)–1	8.01	8.15	8.11	8.04
6–2(4,1)–1	8.22	8.89	8.50	8.52
6–2(4,2)–1	8.07	8.76	8.33	8.34
6–2(4,3)–1	8.11	11.22	8.64	8.79
6–2(4,4)–1	8.07	8.68	8.30	8.34
6–2(4,5)–1	8.17	8.89	8.35	8.42
6–2(4,6)–1	8.13	8.50	8.28	8.28
6–2(5,1)–1	7.90	9.26	8.24	8.38
6–2(5,2)–1	7.91	8.50	8.25	8.25
6–2(5,3)–1	8.07	8.79	8.28	8.32
6–2(5,4)–1	8.05	8.56	8.36	8.33
6–2(5,5)–1	7.94	8.82	8.40	8.37
6–2(5,6)–1	8.12	8.52	8.23	8.30
6–2(6,1)–1	8.20	8.91	8.40	8.47
6–2(6,2)–1	8.09	8.64	8.22	8.25
6–2(6,3)–1	7.90	8.80	8.40	8.40
6–2(6,4)–1	8.11	8.69	8.27	8.30
6–2(6,5)–1	8.09	9.07	8.35	8.45
6–2(6,6)–1	8.09	8.57	8.23	8.26
6–3(1,3,6)–1	8.17	8.79	8.25	8.35
6–3(3,1,6)–1	8.13	8.76	8.23	8.26
6–3(6,3,1)–1	8.07	8.85	8.27	8.33

^1^The network structure is denoted as: input layer nodes—hidden layers (nodes in each hidden layer)—output layer nodes. The input layer nodes are N intake (NI), diet N concentration (DNC), milk yield (MY), forage proportion (FP), live weight (LW) and diet metabolizable energy concentration (DMEC). The output layer node is manure N (MN).

^2^*RRMSE* relative root mean square error (obtained by tenfold cross validation).

To obtain the optimal parameters for ANN models, in the present study, the RRMSE values were tested and compared using same features but different training algorithms, learning rate and threshold for the partial derivatives of the error function as stopping criteria (Table 3). As shown in Table 3, reducing learning rates and thresholds decreased RRMSE when ANN models were trained by both backpropagation and resilient backpropagation with weight backtracking algorithms. In the present study, the resilient backpropagation with weight backtracking algorithm was selected to train the final ANN model because backpropagation algorithm took too much longer time (single-digit minutes vs. more than one hour, data not shown) to train the ANN model than time required for the resilient backpropagation with weight backtracking algorithm. This is consistent with findings reported by Zhao et al.^24^ found that resilient back propagation algorithms took less time to train ANN model for prediction of soil texture distributions. Moreover, based on cross validation procedures, higher prediction accuracy (RMSE) was obtained by models trained with backpropagation algorithm. The results found that reducing values of multiplication factors for the upper and lower learning rate (defined by function *learningrate.factor*) decreased the RRMSE of the mean actual MN. However, the lower RRMSE (8.75%) were observed when turning the parameter of learningrate.factor as list (minus = 0.4, plus = 1.2), which was then selected as values of learningrate.factor in the training of models. The results indicate that although selection of training algorithms did not greatly improve the performance of ANN model, the time consumed for training the model was affected greatly, which needs to be considered in the turning operation.

**Table 3:** Turning of ANN models for manure N output with selected features.

Algorithm	Learning rates^a^	Threshold^b^	RRMSE^c^
Backpropagation	0.01	0.05	8.92^a^
	0.001	0.05	8.60^b^
	0.0005	0.05	8.61^b^
	0.0001	0.05	8.63^b^
	0.00005	0.05	8.59^b^
	0.00001	0.05	8.57^b^
	0.00001	0.01	8.47^c^
Resilient backpropagation with weight backtracking	Minus = 0.5, plus = 1.2	0.01	9.19^a^
	Minus = 0.5, plus = 1.5	0.01	8.98^a^
	Minus = 0.4, plus = 1.2	0.01	8.75^b^
	Minus = 0.3, plus = 1.2	0.01	8.76^b^
	Minus = 0.3, plus = 1.1	0.01	8.76^b^

The features selected are N intake (NI), diet N concentration (DNC), milk yield (MY), forage proportion (FP), live weight (LW) and diet metabolizable energy concentration (DMEC).

^a^Learning rate is a numeric value specifying the learning rate used for backpropagation algorithm. For resilient backpropagation with weight backtracking algorithm it’s a vector or a list containing the multiplication factors for the upper and lower learning rate and defined by function *learningrate.factor.*

^b^Threshold for the partial derivatives of the error function as stopping criteria.

^c^*RRMSE* relative root mean square error (obtained by tenfold cross validation). ^a,b^ Means within a column with different superscripts differ (*P* < 0.05). The significance was determined by one-way analysis of variation and followed by Tukey’s Honest Significant Difference (HSD) test (n = 10, α = 0.05).

Table 4 shows the predictive performance of ANN models affected by reduction of features with all tunable parameters setting to the same values. The NI was the most important feature among the 6 features as the RRMSE increased considerably from 8.48 to 12.6% when NI was excluded from the features list. It suggests that changes of model performance caused by reduction of features might use as an alternative tool for identifying important features. Although the prediction error of ANN model reduced when FP or DNC was excluded from the features, ANN models fitted with the 6 features selected based on the VIF technique had lower SD value, indicating that the ANN model fitted using the 6 features had lower prediction errors within the whole dataset from low to high range of MN excretion.

**Table 4:** Influence of features selected on the ANN model performance.

Features^1^	RRMSE^2^	SD^3^
NI + LW + MY + FP + DNC + DMEC	8.48^b^	0.53
LW + MY + FP + DNC + DMEC	12.6^a^	1.08
NI + MY + FP + DNC + DMEC	8.49^b^	0.80
NI + LW + FP + DNC + DMEC	8.62^b^	0.64
NI + FP + DNC + DMEC	8.84^b^	0.65
NI + LW + MY + FP + DNC	8.76^b^	0.73
NI + LW + MY + FP + DMEC	8.46^b^	0.89
NI + LW + MY + DNC + DMEC	8.45 ^b^	0.89
NI + LW + MY + DNC	8.99^b^	0.56
NI + LW + MY + FP	8.95^b^	0.62
NI + LW + MY + DMEC	8.68^b^	0.49

^1^The learning algorithm = resilient backpropagation with weight backtracking; learningrate.factor = list (minus = 0.4, plus = 1.2). *NI* N intake; *DNC* diet N concentration; *MY* milk yield; *FP* forage proportion; *LW* live weight; *DMEC* diet metabolizable energy concentration.

^2^ *RRMSE* relative root mean square error (obtained by tenfold cross validation). ^a,b^ Means within a column with different superscripts differ (*P* < 0.05). The significance was determined by one-way analysis of variance and followed by Tukey’s Honest Significant Difference (HSD) test (n = 10, α = 0.05).

^3^*SD* standard deviation.

Turning operation can help to find optimal learning parameters for ANN model and achieve its best performance for a considered dataset and selection of learning rate has great influence on model performance^25^. In this study, however, only small performance gain is achieved by lower learning rate (Table 3). On the other hand, variation in features does not affect performance of model greatly (Table 4) when the most important feature was included in the features list. It implies that in general the ANN model is not critically sensitive to the variation in learning parameters.

### New ANN models developed using the combined data

Since the above comparison indicates that the ANN model performed better than the MLR model, new ANN models for MN excretion for dairy cows were developed using the combined data of the present training and testing datasets. Two ANN prediction models were developed with the first one using NI as the primary predictor (Tables 5 and 6) and the second one using LW and MY as primary predictors (Tables 7 and 8) as NI data are not always available especially in commercial farms. The ANN model based on NI had 2 hidden layers with 3 neurons in the first layer, and 6 in the second layer. The input layer consists of NI, DNC, MY, FP, LW and DMEC. The optimized weights and biases are shown in Tables 5 and 6. The ANN model based on LW and MY had 2 hidden layers—the first layer with 4, and the second layer with 2 hidden neurons. The selected features were based on the domain knowledge and included LW, MY, DNC, CDMI and DMEC. The optimized weights and biases are shown in Tables 7 and 8. The prediction performances of these two new ANN models, through the cross validation technique, are given in Table 9. The two ANN models showed good predictive performance, with the R^2^ values in the relationships between actual and predicted MN being 0.83 and 0.79 for models based on NI and LW/MY, respectively, and the corresponding RRMSE was 10.9% and 12.1%, respectively, and the corresponding CCC was 0.76 and 0.70, respectively.

**Table 5:** Optimized weights of connections and biases of the input nodes to nodes on hidden layer one of ANN model using NI as the primary predictor based on the combined data of the present training and testing datasets.

	Nodes on hidden layer one		
	1	2	3
**Input nodes**^a^			
NI	0.032	− 1.640	− 1.869
LW	− 0.125	− 1.684	0.474
MY	1.200	1.599	− 1.241
FP	− 0.253	1.344	− 0.490
DNC	− 1.968	0.413	0.456
DMEC	− 0.5738	3.551	− 0.519
Bias to nodes on hidden layer one	− 0.859	− 0.749	0.432

The ANN model is a feed-forward network with 6 input nodes, 2 hidden layers (the first layer with 3, and the second one with 6 hidden neurons).

^a^*NI* N intake; *DNC* diet N concentration; *MY* milk yield; *FP* forage proportion; *LW* live weight; *DMEC* diet metabolizable energy concentration.

**Table 6:** Optimized weights of connections of the nodes on hidden layer one to two and biases to nodes on hidden layer two and output node of ANN model using NI as the primary predictor based on the combined data of the present training and testing datasets.

	Nodes on hidden layer two						Bias to MN
	1	2	3	4	5	6	
**Nodes on hidden layer one**							
1	− 0.528	− 0.024	− 0.881	− 2.922	1.356	− 1.106	–
2	0.044	− 0.134	0.485	− 1.170	1.294	− 0.833	–
3	0.109	− 1.334	0.119	− 1.989	2.426	0.338	–
Output node							
MN	− 1.050	0.436	1.026	2.978	− 1.551	− 0.155	1.118
Bias to nodes on hidden layer 2	0.321	− 0.361	0.166	− 0.367	− 0.604	0.090	–

The ANN model is a feed-forward network with 6 input nodes, 2 hidden layers (the first layer with 3, and the second one with 6 hidden nodes). *MN* manure N.

**Table 7:** Optimized weights of connections and biases of the input nodes to nodes on hidden layer one of ANN model using LW and MY as primary predictors based on the combined data of the present training and testing datasets.

	Nodes on hidden layer one			
	1	2	3	4
**Input nodes**^a^				
LW	0.0001	− 0.567	− 0.165	− 1.961
MY	− 12.200	− 4.362	− 0.251	− 2.137
DNC	− 2.600	3.271	− 1.404	2.035
CDMI	2.499	0.685	− 0.194	− 0.305
DMEC	5.571	4.780	1.625	1.419
Bias to nodes on hidden layer one	0.461	− 1.285	0.082	− 0.286

The ANN model is a feed-forward network with 5 input nodes, 2 hidden layers (the first layer with 4, and the second one with 2 hidden nodes).

^a^*LW* live weight; *MY* milk yield; *DNC* diet N concentration; *CDMI* concentrate dry matter intake; *DMEC* diet metabolizable energy concentration.

**Table 8:** Optimized weights of connections of the nodes on hidden layer 1 to 2 and biases to nodes on hidden layer 2 and output node of ANN model using LW and MY as primary predictors based on the combined data of the present training and testing datasets.

	Nodes on hidden layer two		Bias to MN
	1	2	
**Nodes on hidden layer one**			
1	8.171	− 0.290	–
2	− 1.788	1.386	–
3	8.982	− 1.294	–
4	4.281	− 1.354	–
**Output node**			
MN	− 0.799	1.966	− 0.089
Bias to nodes on hidden layer 2	− 2.607	0.763	–

The ANN model is a feed-forward network with 5 input nodes, 2 hidden layers (the first layer with 4, and the second one with 2 hidden nodes). *MN* manure N.

**Table 9:** Predictive performance of the ANN models for prediction of manure N output using the whole dataset.

Primary predictors	Features^1^	R^2^	RMSE^2^	RRMSE^3^	CCC^4^
NI	NI + LW + MY + FP + DNC + DMEC	0.83	32.1 ± 1.68	10.9 ± 0.44	0.76 ± 0.025
LW and MY	LW + MY + DNC + CDMI + DMEC	0.79	35.2 ± 1.08	12.1 ± 0.47	0.70 ± 0.021

^1^*NI* N intake; *DNC* diet N concentration; *MY* milk yield; *FP* forage proportion; *LW* live weight; *DMEC* diet metabolizable energy concentration; *CDMI* concentrate dry matter intake.

^2^*RMSE* root mean square error (obtained by tenfold cross validation), mean ± standard deviation.

^3^*RRMSE* relative root mean square error (obtained by tenfold cross validation), mean ± standard deviation.

^4^*CCC* concordance correlation coefficients (obtained by tenfold cross validation), mean ± standard deviation.

## Conclusions

The present study compared the prediction performance for manure N excretion of lactating dairy cows using models developed from the multiple linear regression against those from machine learning algorithms. The results indicate that artificial neural network model has better potential to explore animal and dietary factors which influence manure N excretion in lactating dairy cow when compared with the multiple linear regression approach. A hybrid knowledge-based and data driven approach for artificial neural network model was developed and implemented to selecting features in this study. Results indicate that the resilient backpropagation with weight backtracking algorithm is better than backpropagation algorithm for model training. The optimal network using NI as primary predictors to predict manure N excretion in lactating dairy cows was a feed-forward network with 6 input nodes, 2 hidden layers (the first layer with 3, and the second one with 6 hidden neurons). The alternative network using LW and MY as primary predictors to predict manure N excretion from dairy farm was a feed-forward network with 5 input nodes, 2 hidden layers (the first layer with 4, and the second one with 2 hidden neurons). While currently there is no standard approach to determine optimal set of parameters for those learning parameters in advance, results obtained indicate that the ANN models developed are not critically sensitive to the variation in learning parameters setting. Consequently, two artificial neural network models for prediction of manure N excretion of dairy cows were developed using either N intake or live weight and milk yield as primary explanatory variables. These models provide a novel and useful tool for prediction and mitigation of manure N excretion of dairy cows under typical farming condition managed within grassland-based dairy systems.

## Materials and methods

All the experiments were conducted at the Agri-Food and Biosciences Institute (AFBI) farm at Hillsborough, County Down, UK. All the experiments and procedures complied with the requirements of the UK Animals (Scientific Procedures) Act 1986 and were approved by the AFBI Hillsborough Ethical Review Group. All the experiments were performed in accordance with relevant guidelines and regulations (following the ARRIVE guidelines^26^).

### Data description

Data used were collated from 43 total diet digestibility studies with 951 lactating dairy cows undertaken at Agri-Food and Biosciences Institute in Northern Ireland over a period of 26 years (1990–2015). The data from studies undertaken between 1990 and 2002 were used as the training dataset (n = 564) and undertaken between 2005 and 2015 as the testing dataset (n = 387). The training data were used to develop prediction models for MN using MLR and the three selected machine learning algorithms (ANN, RFR and SVR). These new models were then tested for their predictive performance using the training dataset by tenfold cross validation. The testing dataset were used for the independent evaluation and comparison of predictive ability of different modeling approaches. The information of the two datasets on numbers of experiments, cow genotypes and forage types offered are presented in Table 10. Data on live weight, milk production, feed intake, N intake and outputs are presented in Table 11. The datasets used in the present study showed a various cow genetic merit and a broad range in LW (379–781 kg), MY (5.1–40.2 kg/d), total dry matter intake (7.54–26.6 kg/d), FP (0.21–1.00%), DNC (19.0–38.0 g/kg DM), diet metabolizable energy concentration (DMEC, 9.68–19.4 MJ/kg DM) and NI (155–874 g/d), which represents typical dairy production conditions managed within grassland-based dairy systems in the West and North Europe.

**Table 10:** Information on experiment, animal and forage types in the training and testing datasets of dairy cows used in the present study.

	Training dataset	Test dataset
Years of experiments	1990–2002	2005–2015
Number of experiments	27	16
Number of individual cow data	564	387
**Cow breeds**		
Holstein–Friesian	534	269
Others^a^	30	118
Forage types^b^	GS, FG	GS, MS, WCW

^a^Including Holstein crossbreds, Norwegian and Swedish Red.

^b^*GS* grass silage; *FG* fresh grass; *MS* maize silage; *WCW* whole crop wheat silage.

**Table 11:** Descriptive statistics of animal, dietary and nitrogen utilization variables in the present study.

Features^1^	Unit^3^	Abbreviation	Training dataset				Test dataset			
			Mean	SD^2^	Minimum	Maximum	Mean	SD^2^	Minimum	Maximum
Live weight	kg	LW	564	65.3	385	781	549	70.1	379	757
Milk yield	kg/d	MY	21.4	6.61	6.10	49.1	23.6	7.16	5.87	48.8
Energy-corrected milk yield	kg/d	ECMY	21.8	6.70	5.53	45.6	24.0	6.50	5.10	49.5
Forage DMI	kg/d	FDMI	9.33	2.79	2.96	18.9	10.0	2.80	3.60	16.8
Concentrate DMI	kg/d	CDMI	7.08	3.51	0	16.9	8.18	2.99	3.21	16.0
Total DMI	kg/d	TDMI	16.4	3.02	7.54	24.3	18.2	2.92	10.8	26.6
Forage proportion	kg/kg DM	FP	0.58	0.183	0.21	1.00	0.55	0.142	0.31	0.79
Diet N concentration	g/kg DM	DNC	29.3	4.15	17.0	43.3	27.8	4.02	18.0	43.0
Diet ME concentration	MJ/kg DM	DMEC	12.1	0.92	9.89	19.1	12.1	0.82	9.68	14.1
N intake	g/d	NI	486	129.6	155	874	506	106.6	228	798
Feces N output	g/d	FN	142	36.1	48.4	241	159	32.9	73.7	284
Urine N output	g/d	UN	209	69.1	69.6	452	178	61.4	44.7	364
Manure N output	g/d	MN	351	97.7	130	679	337	77.1	159	577

^1^*DMI* dry matter intake; *ME* metabolizable energy; *N* nitrogen.

^2^standard deviation.

^3^*DM* dry matter.

### Digestibility measurements

Cows were housed in free-stall cubicle accommodation for at least 20 d before commencing digestibility trials in metabolism units for 8 d with feed intake, milk production and feces and urine collected during the final 6 d. Throughout the whole experiment, cows were offered experimental diets ad libitum and had free access to water. During the final 6 d, the following measurements for each individual cows were carried out to generate total digestibility data used in the present study. Forages and concentrates offered and refused were recorded daily and sampled for analysis of feed dry matter (DM), N concentration and forage proportion. Feces and urine outputs were collected daily and sampled for DM (feces only) and N concentration. Milk yield was recorded daily and sampled for analysis fat, protein and lactose concentrations. Live weight was measured on the first and last days in the metabolism unit. Details in feed intake, feces and urine collection and methods used for analysis of feed, feces, urine and milk samples were described by Yan et al.^6^.

### Data preprocessing

#### Normalization of input data for ANN model

Because features (variables) in raw data may have different dynamic ranges, which may result in poor model performance, it is recommended to normalize them to make ANN training more efficient by performing normalization process for the raw inputs^10^. In the present study, all the input data for ANN models were normalized into the interval [0, 1] by performing Min–Max normalization technique^27^ using Eq. (1):

1 $$X_{norm} = \frac{{X {-} X_{min} }}{{X_{max} - X_{min} }}$$

where *X*_*norm*_ or *X* is the normalized or original value, *X*_*min*_ or *X*_*max*_ is the minimum or maximum values of the input data.

After finding the optimal tuning parameter, all normalized data for MN obtained by ANN models were denormalized into their original scale using Eq. (2) ^27^:

2 $$Y = Y_{norm} * \, \left( {Y_{max} - \, Y_{min} } \right) \, + \, Y_{min}$$

where *Y*_*norm*_ or *Y* is the normalized or demoralized value, *Y*_*min*_ or *Y*_*max*_ is the minimum or maximum values of the output data.

#### Knowledge-based and data driven feature selection

Feature selection is an essential step during development of models, which can hugely impact the generalization and predictive ability of models^10,28^. In the present study, a hybrid knowledge-based and data driven approach was developed and implemented to selecting features. Knowledge in animal science and the process of digestibility trial were applied to diagnosing and removing irrelevant features before the implementing of data driven feature selection process. For instance, the features of feces N output (FN) and urine N output (UN) were excluded from the set of features in present study according to prior background and expert knowledge. Because the data of UN and FN were obtained from analyzing urine and feces samples and then they were summed up and treated as new feature MN, both FN and UN are heavily correlated with MN. Their inclusion in the features list might cause poor generalization performance of the models. Furthermore, the optimal features selected from data driven approach may need to be diagnosed based on background knowledge in animal science according to the scenarios of model application. For instance, several variables (e.g. NI and FP) included in datasets used in this study may not be available in commercial farms. Therefore, alternative feature (concentrate dry matte intake, CDMI) was selected and included into the feature list in this study based on the domain knowledge and then new ANN model suits for commercial farms was developed.

The filter method was applied for feature selection using the Pearson correlation matrix and variance inflation factor (VIF) technique. The first step was to use the Pearson correlation matrix to identify features which might correlate each other for prediction of MN excretion, because using correlated features in models could influence performance of these models with a biased outcome. If two features were heavily correlated, the less important one was removed from the set of features to minimize adverse effects on model performance. Afterwards, the VIF analysis was applied to detect multicollinearity, which has been widely used as a measure of the degree of multicollinearity among input features. A VIF score was calculated for each feature and those with high values were removed. The threshold score for the VIF analysis was 5 and features with a VIF score below this threshold were selected. The VIF score was computed by VIF function in R^29^.

#### Modelling and analysis using the training dataset

In the present study, four models based on the MLR ANN, RFR and SVR were developed using the training dataset and these new models were tested using the testing dataset for comparison of their prediction performance for MN outputs in lactating dairy cows (presented later). The MLR with the stepwise procedure for selection of independent variables was used as benchmark model since it is a well-known technique and has been applied for modelling in a wide range of applications in animal science research. Alternative modeling approaches proposed in the present study were ANN, RFR and SVR. To compare the performance, models developed with different approaches and ensure that the same resampling sets were used between calls, the same random number seeds were set prior to perform the process of training, fitting and testing models. All statistical analyses were performed with R^29^.

### Multiple linear regression

The MLR model (Eq. 3) selected in the present study for the prediction of MN output was published in 2006^6^ which was developed using the same training dataset listed in Table 2. To improve the estimation of the regression parameters, experiment was included as a random factor during the development of MLR model. The dataset had a large range within each dependent or independent variable, e.g., MN, NI, LW, MY, FP and DNC, which is vital to ensure the development of robust regression model applicable under various farming conditions^10^.

3 $${\text{MN }}\left( {{\text{g}}/{\text{d}}} \right) \, = \, 0.{\text{749 NI }} + \, 0.0{\text{65 LW }}{-}{ 1}.{\text{515 MY }}{-}{ 17}.0$$

where NI, LW and MY are N intake (g/d), live weight (kg) and milk yield (kg/d), respectively.

### Artificial neural networks

In the present study, ANN was fitted using R package *neuralnet* which was built to train neural networks in the context of regression analyses. The details of ANN training and application of *neuralnet* were described by Günther and Fritsch^30^. Multilayer perceptron networks trained with backpropagation learning algorithms were used and consist of an input layer, hidden layer(s) and an output layer. The input variables were obtained by using the feature selection algorithm described in the section ‘Knowledge-based and data driven feature selection’, and the neuron in output layer represents MN. The ANN models were trained based on the selection of training algorithms and learning parameters including the number of hidden layer(s), number of neurons in hidden layer(s), error function, threshold for partial derivatives of the error function as stopping criteria, and activation function etc.. The optimized number of hidden layer(s), number of neuron(s) in the hidden layer(s), learning algorithms, learning rate and other learning parameters were obtained on the basis of prediction performance measured as relative root mean square error (RRMSE, Eq. 6) with tenfold cross validation and then the best topology/architecture was finalized.

### Random forest regression

The RFR is an ensemble machine learning method and a nonparametric technique derived from classification and regression trees which are constructed using a bootstrap aggregating (bagging) method from the training data^31^. In RFR, prediction is conducted by averaging the individual tree predictions. A detailed description of RFR theory can be found in the report by Breiman^32^. The RFR was implemented by the *randomForest* function in the R package (version 3.6.1). To select the optimal hyperparameters for learning algorithm, tuning process was performed based on the R package ranger. The hyperparameters include number of trees to grow (*ntree*), number of randomly drawn candidate variables (*mtry*), sample size and node size. Grid search strategy was used to choose the candidate hyperparameter values and the performances of the trained algorithm with different values of the hyperparameters were evaluated as RRMSE (Eq. 6) by using tenfold cross validation.

### Support vector regression

The SVR uses similar principles as support vector machine, a supervised non-parametrical statistical learning technique that uses the kernel functions and the maximum margin algorithm to solve the nonlinear problem^33^. The detailed theoretical background and description of SVR can be found in the report by Cristianini and Shawe-Taylor^34^. The SVR model performs the regression estimation by risk minimization where the risk is measured by a loss function. In this study, R package e1071 was used and the *svm* function was implemented to fit SVR model. The radial basis kernels function, the most commonly used kernels types, was employed in training and predicting process. Parameter tuning was performed by using grid search over supplied parameter ranges and the best combination of parameters (lowest RMSE) were selected. The performance of SVR model was measured as RRMSE (Eq. 6) with tenfold cross validation.

### Criteria selected to access model prediction performance

The MLR model and the three new models (ANN, RFR and SVR) was developed and compared in terms of their prediction performance for MN outputs in lactating dairy cows based on the datasets listed in Table 2. The predictive performance of models were evaluated using coefficient of determination (R^2^), root mean square error (RMSE), relative root mean square error (RRMSE) and concordance correlation coefficient (CCC), based on the actual and predicted values. The R^2^ was calculated using Eq. (4). The RMSE and RRMSE were produced in a tenfold cross validation process (10 RMSE data generated) using Eq. (5)^35^ and Eq. (6)^36^, respectively. The concordance correlation coefficient (CCC), a further measure of the agreement between observed and predicted values, was given by Eq. (7)^37^. The tenfold cross validation was used to evaluate prediction performance of these models (MLR, ANN, RFR and SVR)The obtained RMSE, RRMSE and CCC values (n = 10) through the tenfold cross validation were compared among the 4 models using one-way analysis of variance and then followed by Tukey’s honest significant difference (HSD) test (α = 0.05). The same cross validation folds were used for all modeling scenarios to compare cross all of the models performance.

4 $$R^{2} = 1 - \frac{{\sum \left( {y_{i} - \hat{y}} \right)^{2} }}{{\sum \left( {y_{i} - \overline{y}} \right)^{2} }}$$

5 $$RMSE = \sqrt { \frac{1}{n}\mathop \sum \limits_{i = 1}^{n} \left( {y_{i} - \hat{y}} \right)^{2} }$$

6 $$RRMSE = (RMSE/\overline{y}) \times \, 100$$

7 $$CCC = \frac{{2 \cdot \,r \cdot \,S_{{\widehat{y}}} \cdot\,S_{y} }}{{S_{{\widehat{y}}}^{2} + S_{y}^{2} + \left( {\mathop \sum \nolimits_{i = 1}^{n} \frac{{\left( {y_{i} - \widehat{y}} \right)}}{n}} \right)^{2} }}$$

where $y_{i}$ is actual MN, $\widehat{{y_{i} }}$ is predicted MN, $\overline{y}$ is the mean of actual MN and *n* is the number of observations, *r* is the Pearson correlation coefficient between $\widehat{{y_{i} }}$ and $\overline{y}$, $S_{{\hat{y}}}$ and $S_{y}$ are the respective standard divisions.

## Data Availability

The datasets used and/or analyzed during the current study are available from the corresponding author on reasonable request.
